# A multi-scale robotic tool grasping method for robot state segmentation masks

**DOI:** 10.3389/fnbot.2022.1082550

**Published:** 2023-01-10

**Authors:** Tao Xue, Deshuai Zheng, Jin Yan, Yong Liu

**Affiliations:** School of Computer Science and Engineering, Nanjing University of Science and Technology, Nanjing, Jiangsu, China

**Keywords:** human-robot collaboration, instance segmentation, robotic grasping, grasp detection, robotic grasp platform

## Abstract

As robots begin to collaborate with humans in their daily work spaces, they need to have a deeper understanding of the tasks of using tools. In response to the problem of using tools in collaboration between humans and robots, we propose a modular system based on collaborative tasks. The first part of the system is designed to find task-related operating areas, and a multi-layer instance segmentation network is used to find the tools needed for the task, and classify the object itself based on the state of the robot in the collaborative task. Thus, we generate the state semantic region with the “leader-assistant” state. In the second part, in order to predict the optimal grasp and handover configuration, a multi-scale grasping network (MGR-Net) based on the mask of state semantic area is proposed, it can better adapt to the change of the receptive field caused by the state semantic region. Compared with the traditional method, our method has higher accuracy. The whole system also achieves good results on untrained real-world tool dataset we constructed. To further verify the effectiveness of our generated grasp representations, A robot platform based on Sawyer is used to prove the high performance of our system.

## 1. Introduction

With the increasingly serious aging of the population, how to provide effective homecare for the growing elderly population has ushered in new challenges and changes, especially the COVID-19 epidemic, which makes the need for homecare for the elderly extremely urgent. In order to prevent the elderly from using tools incorrectly and to ensure the safety of tools when using them, we effortlessly draw on our understanding of the functions that tools and their parts provide. Using vision, we can identify the function of the part, so we can find the right tool part for our operation. As robots like PR2, Asimo, and Baxter begin to collaborate with humans in homecare industry, they will also need us to have a more detailed understanding of the tools involved in the task.

When completing tasks through human-robot collaboration, robots are designed to provide more assistance to humans, rather than let the robot perform all tasks autonomously. There are two reasons for this. Firstly, the and level of knowledge and the training required for robots to complete tasks on their own are difficult to establish and collect. Secondly, despite the significant progress made in robotics such as manipulation (Kroemer et al., 2015; Fu et al., 2016), robots are still far from having the fine manipulation capabilities required for tasks such as furniture assembly (for example, using a screwdriver on small screws). Therefore, we hope that the robot can choose the behavior suitable for the robot, while letting the human worker perform the action more suitable for the human. For example, robots may provide supportive or transmit behaviors, such as stabilizing components or bringing heavy components required for assembly (Mangin et al., 2017), while human workers can perform operations that require more adaptability to tasks, such as screwing screws. Therefore, in the task of using various tools through human-robot collaboration, how to understand the task requirements and assign them to different states of robots and humans to grasp tools is a very critical issue.

Brahmbhatt et al. (2019) used thermal camera to study human grasping contacts on 50 household objects textured with contact maps for two tasks. Fang et al. (2020) developed a learning-based approach for task-oriented grasping in simulation with reinforcement learning. Liu et al. (2020) considered a broad sense of context and proposed a data-driven approach to learn suitable semantic grasps. These methods are able to solve the problem of understanding task requirements related to grasp tools through pixel-level enlightening segmentation of a small group of known object categories (Do et al., 2018). However, for collaborative tasks, there is still a lack of consideration for different states that lead to different tool grasping representation. In order to realize the understanding of tools according to different state definitions of robots, we constructed a tool classification dataset used to analyze the different states played by robots when grasping various tools.

We recruited some volunteers to take on different states in grasping the tools in the dataset. And we recorded the grasping areas corresponding to different states and counted these positions. We borrowed the idea of region classification and proposed the state semantics (grasp and handover) region, that is, different states often make people grasp different position of tools. Based on the knowledge of this region, we define two types of robot states: active operator and assisting passer, corresponding to the previous semantics “grasp” and “handover.”

The main contributions of our work mainly include the following four points:

1. We proposed a modular system for multi-states tool grasping task under human-robot interaction, which can realize the collaborative grasping and interaction of humans and robots based on tasks.
2. A multi-layer instance segmentation network is proposed to complete the classification of operating areas for task-related tools. Therefore, in different tasks, we can find the most suitable grasping area for humans or robots in different states.
3. Considering that grasping based on the local semantic region of the tool will increase the variation range of the receptive field, we propose a multi-scale grasping convolutional network MGR-Net based on state semantics to improve the prediction accuracy of the network.
4. We collected real-world tool images through “realsense” camera as a test set, and the experimental results show that our method performs well on untrained real-world tool images. Furthermore, we used robotic platform based on Sawyer to validate our grasping representation.

The other chapters of this article are arranged as follows. In Section 2, we briefly review related literature. In Section 3, we detail the proposed grasping framework based on semantic state area. In Section 4, Our experimental results are presented. Finally, we conclude this work in Section 5.

## 2. Related work

Learning to use an item as a tool requires an understanding of what it helps to achieve, the properties of the tool that make it useful, and how the tool must be manipulated in order to achieve the goal. In order to further meet the operational requirements of our robots based on different states, the tool grasping tasks under different states can be divided into the following three aspects:

1. Detection of tools related to different tasks.
2. Research on the properties of the tool itself.
3. Robotic grasping detection of tools.

### 2.1. Task-related tool detection

The earliest classification of tasks is mostly to find corresponding task objects in multiple objects. With the great power of machine learning in classification, researchers find that novel objects grasp detection can be classified into two parts, which is graspable or ungraspable. SVM has been widely used in grasp feature classification (Fischinger et al., 2015; Ten Pas and Platt, 2018). Ten Pas and Platt (2018) used knowledge of the geometry of a good grasp to improve detection. Through sampling lots of hand configuration as the input features, they used the notion of an antipodal grasp to classify these grasp hypotheses. Deep learning methods are also been applied in grasp detection. Lenz et al. (2015) presented a two-step cascaded system with two deep networks and ran at 13.5 s per frame with an accuracy of 93.7%.

In order to better identify task-related tools among multiple types of tools and avoid the interference of irrelevant tools, instance segmentation methods are introduced to achieve more accurate tool detection accuracy. Top-down methods (He et al., 2017; Chen et al., 2020) solve the problem from the perspective of object detection. For example, first detecting an object, then segmenting it in the box. Recently, the anchor-free object detectors were used by some researchers and got good results (Tian et al., 2019). Bottom-up methods (Liu et al., 2017; Gao et al., 2019) view the task as a label-then-cluster problem. These method learn the per-pixel embeddings and then cluster them into groups. The latest direct method (SOLO) (Wang et al., 2020a) no longer relies on box detection or embedding learning, and deals with instance segmentation directly. Wang et al. (2020b) appreciate the basic concept of SOLO and further explore the direct instance segmentation solutions.

### 2.2. Tool attribute classification

The above methods can identify objects of known classes very well. However, in the case of using a spoon, the robot needs to know which part of the spoon to grasp and which part to hold the soup. Work on grasp affordances tends to focus on robust interactions between objects and the autonomous agent. It is typically limited to a single affordance per object. Moreover, affordance labels tend to be assigned arbitrarily instead of through data-driven techniques to collect human-acceptable interactions about grasping. Krüger et al. (2011) focus on relating abstractions of sensory-motor processes with object structures [e.g., object-action complexes (OACs)], and capture the interaction between objects and associated actions through an object affordance. Others use purely visual input to learn affordances to relate objects and actions through deep learning or supervised learning techniques (Hart et al., 2015). Chu et al. (2019) presented a novel framework to predict the affordance of objects *via* semantic segmentation.

It is worth considering that in the interactive use of tools, robots not only need to find the task-related tools and operating areas, but also clarify the state of the robot at this time, whether it is the “leader” or the “assistant” of the task. However, the previous classification of tool attributes at this time is not sufficient to meet this goal, they only consider the case where the robot is a single operator. In order to solve this problem, based on the attributes generated by the classification of tool functions, we focus on the grasping operation during interactive tasks. Through data-driven technology, the functional attributes of the tool are combined with the state of the robot to find the optimal grasping area of the tool for the robot under different states.

### 2.3. Robotic grasping detection

Deep learning has been a hot topic of research since the advent of ImageNet success and the use of GPU's and other fast computational techniques. Also, the availability of affordable RGB-D sensors enabled the use of deep learning techniques to learn the features of objects directly from image data. Recent experimentations using deep neural networks (Schmidt et al., 2018; Zeng et al., 2018) proved that they were quite efficient when calculating stable grasp configurations. Guo et al. (2017) fused tactile and visual data to train hybrid deep architectures. Mahler et al. (2017) trained a Grasp Quality Convolutional Neural Network (GQ-CNN) with only synthetic data from Dex-Net 2.0 grasp planner dataset. Levine et al. (2018) presented a method for learning hand-eye coordination for robotic grasping from monocular images. They use a CNN for grasp success prediction, and a continuous servoing mechanism used this network to continuously control the manipulator. Antanas et al. (2019) proposed a probabilistic logic framework that is said to improve the grasping capability of a robot with the help of semantic object parts. This framework combines high-level reasoning with low-level grasping. The high-level reasoning leverages symbolic world knowledge through comprising object-task affordances, categories, and task-based information while low-level reasoning depends on visual shape features.

Most of these grasp synthesis approaches are enabled by representing the grasp as an oriented rectangle in the image (Dong et al., 2021). Kumra et al. (2020) used an improved version of grasp representation, complemented by a novel convolutional network, which improves the accuracy of robotic grasping. Depierre et al. (2021) introduced a new loss function, which associates the regression of the grab parameters with the score of the grabability. Dong et al. (2022) used the transformer network as an encoder to obtain global context information. Shukla et al. (2022) proposed GI-NNet model based on inception module, it can achieve better results under limited data sets, but it is less adaptable to big data. These grasping methods tend to focus on the tool itself, ignoring the impact of different tasks on grasping. Especially in human-computer interaction tasks, different states prompt the robot to grasp different parts of the tool. In order to solve the problem of robot grasping under human-computer interaction, we modified the grasping representation of the tool based on the different state semantic regions of the tool. Through an improved grasping neural network, the accuracy of grasping detection is improved.

## 3. Method

**Figure 1:**
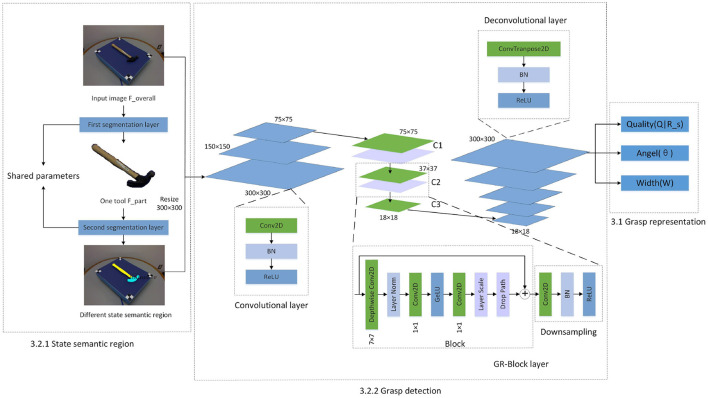
Our MGR-Net based on state semantic regions.

In this human-robot collaboration work, we consider the operating area of the tool when people are in the two different states of leader and assistant. And let our network learn this selection rule, so that when the robot assists the human or the robot operates under the guidance of the human, it can find the relevant task position as much as possible. In this paper, in order to study how to generate the robot grasp detection problem under different states, the following state semantic region classification and grasping detection framework of collaborative task are proposed, as shown in the Figure 1.

Our grasping detection network mainly consists of two parts. First, finding the task-related state semantic region of object. Second, finding the most suitable grasp configuration for robots or humans based on different state semantic regions.

### 3.1. Grasp representation

In this work, we define the robot grasping detection problem as predicting unknown objects from the n-channel image of the scene and assigning states based on the task according to the provided task description, so as to carry out the corresponding grasping and execute it on the robot. Instead of the five-dimensional grip representation used in Kumra and Kanan (2017), we use an improved version similar to the grasp representation proposed by Morrison et al. (2020). Considering that the optimal grasping configuration of the robot will change in different state states, we incorporate the attribute of the state semantic area into the robot frame, and change the grasping posture to be expressed as:


(1) $$\begin{array}{l} G=\left(P,\theta ,W,Q|R_{s}\right) \end{array}$$

Among them, *P* = (*x, y, z*) is the center position of the tool tip, θ is the rotation of the tool around the z-axis, *W* is the required width of the tool, *R*_*s*_ represents the state semantic area, and *Q*|*R*_*s*_ represents the grasp score of the corresponding state area.

The grasp quality score *Q* is the grasp quality of each point in the image, and is expressed as a fractional value between 0 and 1, with values closer to 1 indicating a greater chance of successful grasping. θ represents a measure of the amount of angular rotation at each point required to grasp the object of interest, expressed as a value in the range [$\frac{-\pi }{2}$, $\frac{\pi }{2}$]. *W* is the desired width, expressed as a measure of uniform depth, and expressed as a value in the range [0, *W*_*max*_] pixels. *W*_*max*_ is the maximum width of the gripper.

### 3.2. Grasp detection network

#### 3.2.1. State semantic region

**Figure 2:**
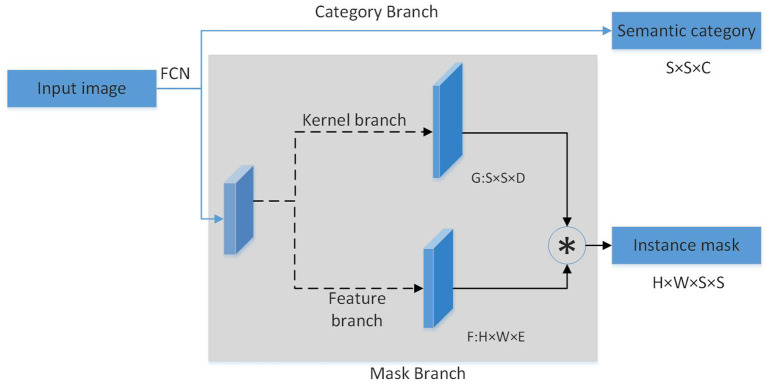
State semantic region segmentation.

We input image *F*_*overall*_ to the first layer of tool segmentation network. Through the generated mask, we construct the input image *F*_*part*_ of the second layer of state semantic segmentation network. Based on the state that the robot assumes in the task, the second layer finally generates semantic regions related to the robot state. More descriptions of the tool datasets will be introduced in Section 4.1. The modules in the segmentation layer are shown in Figure 2.

Two segmentation layers is designed to achieve different purposes. The first layer of the overall segmentation layer finds out the mask of the task-related object in the multi-object environment, which includes two branches: (1) Category Branch is responsible for predicting the semantic category of the object. (2) Mask Branch is responsible for predicting the mask region of the object. The second layer further divides the task object based on the state to obtain the state semantic area of the object. The state semantic area mainly contained in this layer is the “grasp” area as the state of leader and the “handover” area as the state of assistant. The difference between this layer and the first layer is: (1) Category Branch is responsible for predicting the state semantic category of the task area of the object. (2) Mask Branch is responsible for predicting the mask of the semantic area of different states of the object. Each layer uses FPN behind the backbone network to cope with the size. After each layer of FPN, the above two parallel branches are connected to predict the category and position. The number of grids in each branch is correspondingly different. Small examples correspond to more grids.

Category Branch is responsible for predicting the semantic category of each task area of the object. Each grid predicts the category S×S×C. The mask branch is decomposed into mask kernel branch and mask feature branch, which correspond to the learning of the convolution kernel and the learning of features, respectively. The output of the two branches is finally combined into the output of the entire mask branch. For each grid, the kernel branch predicts the D-dimensional output, which represents the predicted weight of the convolution kernel, and D is the number of parameters. So for the number of grids of S×S, the output is S×S×D. Mask feature branch is used to learn the expression of features. Its input is the features of different levels extracted by backbone+FPN, and the output is the mask feature of H×W×E, denoted by F.

#### 3.2.2. Grasp detection

Feature output is similar to Kumra et al. (2020), and also contains three different prediction maps (*Q*|*R*, angle, width) represented by the grasping posture, as shown in the Figure 1. But the difference is that since our grasping posture contains the content of the state assignment area, our grasping score is also closely related to the character area.

The input image and the state semantic region mask corresponding to the task are sent to the convolutional layer together. The convolutional layer consists of conv2d layer, batch normalization (BN) layer and relu layer. The output of the convolutional layer is fed to 3 GB-Block layers (C1–C3), the first two GR-Block layer contains a Block and Downsampling, as shown in the Figure 1. We designed this Block from Liu et al. (2022). Three conv2d layers are used in Block with different kernel functions, and Layer Norm replaces Batch Norm for better effect. Since we focus on the semantic area above the object rather than the object itself, the change in the size of the object will increase the difficulty of detection. We use three Block of different sizes to obtain different receptive fields to improve the detection accuracy. A downsampling module is to connect two Block of different sizes, as shown in the Figure 1. After that, in order to more easily interpret and preserve the spatial characteristics of the image after the convolution operation, we use five deconvolutional layers to upsample the image. Therefore, we get the same size image at the output as the input. Grasp representation is generated as network output from the deconvolutional layer.

#### 3.2.3. Loss function

For each input image *p*, combined with the local attribute region image *p*_*k*_ generated by its different state semantic regions *M*, our grasping network is optimized by the following loss function:


(2) $$\begin{array}{l} loss\left(G_{k},\hat{G_{k}}\right)=\frac{1}{n}\overset{n}{\underset{i=1}{\sum }}s_{i} \end{array}$$

where *s*_*i*_ is given by:


(3) $$s_{i}=\left\{ \begin{array}{l} 0.5\cdot {\left({\hat{G_{k}}}_{i}-f\left(G_{k}{}_{i}\right)\right)}^{2},\;if|{\hat{G_{k}}}_{i}-f(G_{k}{}_{i})|\;<1\\ |{\hat{G_{k}}}_{i}-f(G_{k}{}_{i})|-0.5\;otherwise \end{array}\right.$$

*G*_*k*_ is the grasp generated by the network corresponding to *p*_*k*_ and $\hat{G_{k}}$ is the ground truth grasp.

## 4. Experiment

We implemented our detection network in PyTorch and the computer configuration used in the experiment is intel core I7-8700 CPU and NVIDIA 2080ti GPU. The following experimental part mainly contains three pieces.

### 4.1. Dataset

In order to meet the image input required by our network, we constructed a dataset of collaboration tools. We selected 6,000 tool images from IIT-AFF Dataset (Nguyen et al., 2017), UMD Dataset (Myers et al., 2014), Cornell Grasp Dataset and Jacquard Grasping Dataset (Depierre et al., 2018). We resize the images in the tool dataset to the same size. This tool dataset is used for two networks. One is mainly used for the classification of the object task area. At this time, 90% of the images in the dataset are used as the training set, and the rest are the test set. Another use is tool grasp detection based on the robot's state. The training set at this time comes from the jacquard part of the tool dataset, there are 4,000 images, and the remaining jacquard images are used as the test set together with other parts of the dataset. The extended version of Cornell Grasp Dataset comprises of 1,035 RGB-D images with a resolution of 640 × 480 pixels of 240 different real objects with 5,110 positive and 2,909 negative grasps. The annotated ground truth consists of several grasp rectangles representing grasping possibilities per object. The Jacquard Grasping Dataset is built on a subset of ShapeNet which is a large CAD models dataset. It consists of 54 k RGB-D images and annotations of successful grasping positions based on grasp attempts performed in a simulated environment. In total, it has 1.1 M grasp examples.

### 4.2. Task area

**Figure 3:**
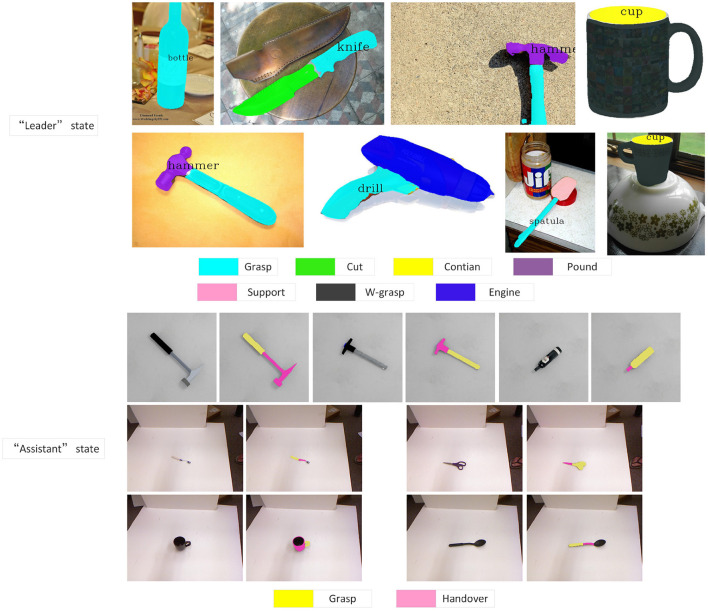
Segmentation results based on “leader” and “assistant” state.

In this section, we mainly discuss the results of semantic region classification. Different states are given to the robot according to the task, and the robot has a more specific functional area classification for the tool. As shown in Figure 3, when the robot acts as the “leader,” the tools are classified according to their affordance. Such classification enables the robot to grasp more accurately, and avoids damage to the object or the gripper caused by the wrong grasping position. When the robot acts as an “assistant,” it always expects the human to grasp the most suitable position for grasping. Therefore, the robot needs to avoid this grasping area as much as possible and find a suitable area for handover. Through the delivery of the robot, human can always grasp the tool most efficiently and safely. For example, when passing scissors, such classification can avoid being accidentally injured by scissors due to people's carelessness.

To further test the effectiveness of our two-layer segmentation network, we compare it with other methods on the IIF-AFF Dataset, as shown in the Table 1. Among them, grasp#2 and handover#2 represent the classification results when the robot is “assistant.” It can be seen that our network still has high accuracy.

**Table 1:** Performance on IIT-AFF dataset.

	**DeepLab (Chen et al.**, **2017****)**	**Affordance-net (Do et al.**, **2018****)**	**RAN-ResNet50 (Zhao et al.**, **2020****)**	**Our method**
Contain	68.84	79.61	80.20	87.10
Cut	55.23	75.68	78.04	72.80
Display	61.00	77.81	79.14	91.20
Engine	63.05	77.50	81.22	85.50
Grasp#1	54.31	68.48	71.59	82.60
Hit	58.43	70.75	88.52	91.00
Pound	54.25	69.57	76.91	81.90
Support	54.28	69.81	80.12	78.90
Grasp#2	–	–	79.27	88.86
Handover#2	–	–	77.96	80.08

### 4.3. Grasp detection metric

In order to better compare our results with the results of previous researchers, we refer to the comparison scale in Jiang et al. (2011) and make some optimizations. Since our grasp is aimed at a smaller task area, we set the iou value between ground truth grasp rectangle and the predicted grasp rectangle to two types: (1) The iou value is >25% for rough grasping. (2) The iou value is >50% for stable and accurate grasping. In addition, The offset between the grasp orientation of the predicted grasp rectangle and the ground truth rectangle is <30^°^.

### 4.4. Grasp detection

**Figure 4:**
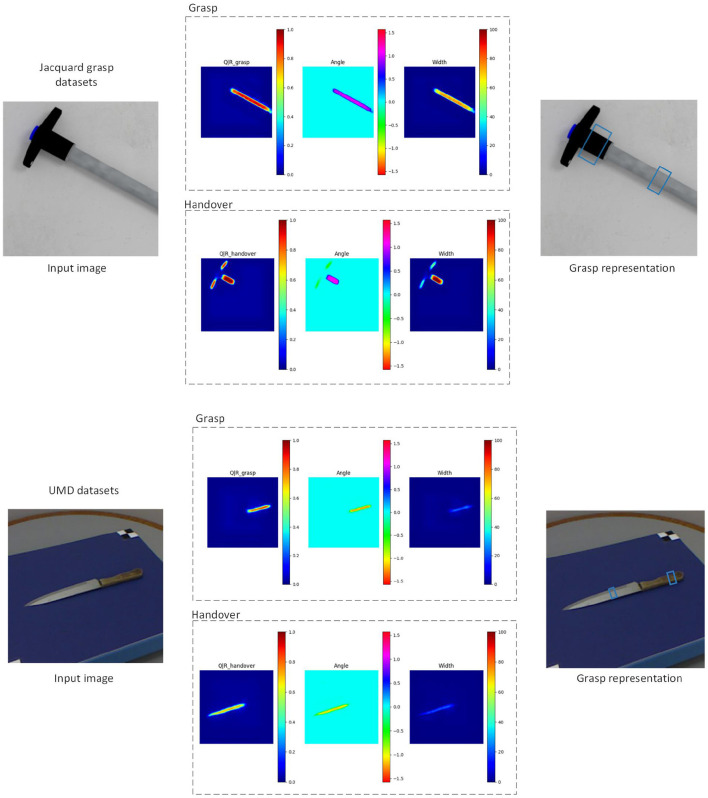
Qualitative results on different datasets.

We discuss the results of our experiments here. We evaluate MGR-Net on our tools datasets, and demonstrate that our model is able to adapt to various types of tool objects. In addition, our method can not only grasp the whole object, but also understand the robot operation information contained in the task and grasp a certain area of the tool, so as to help people safely grasp the target tool. Figure 4 shows the qualitative results obtained on previously unseen tools.

**Figure 5:**
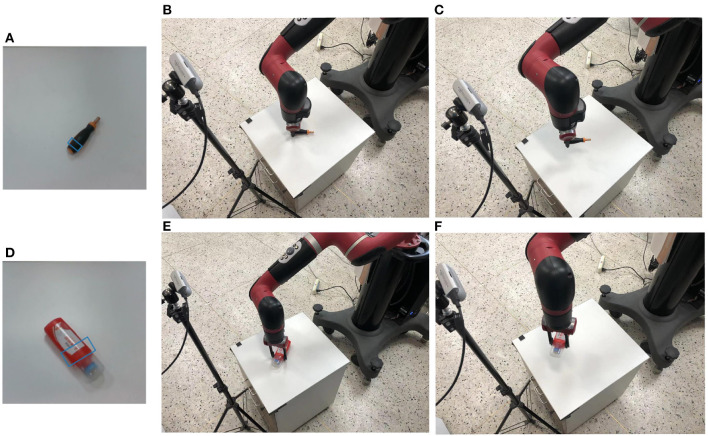
Verification through robot platform. **(A, D)** The results of grasp detection. **(B, E)** The robot grasping tools. **(C, F)** The robot lifting tools to indicate whether the grasping is successful or not.

The Table 2 shows the changes in the overall grasp due to the improvement of the network module. After obtaining the grasping representation of the tool through our detection network. Based on the robot platform, we use Sawyer robot to verify the grasping representation. Since the coordinate relationship between the camera and the robot is known, we transform the grasp representation from the image space to the robot coordinate system. Figure 5 shows the process of our verification through Sawyer robot, where Figures 5A, D are the result graphs generated by our capture of the detection network. After the camera space is converted to the robot space, Sawyer reaches the designated position and closes the gripper, as shown in Figures 5B, E. Figures 5C, F lift the object upward to prove whether our grasp is successful or not. We used 20 unseen real tools. Each test object contains five different positions and directions and the grasp accuracy is 92%. The experiment proves the effectiveness of our method.

**Table 2:** Ablation study.

**Network structure**	**Accuracy (25%)**	**Accuracy (50%)**
Residual block	0.95	0.83
Only block	0.95	0.84
GR-block	0.96	0.87

### 4.5. Comparison of different approaches

**Figure 6:**
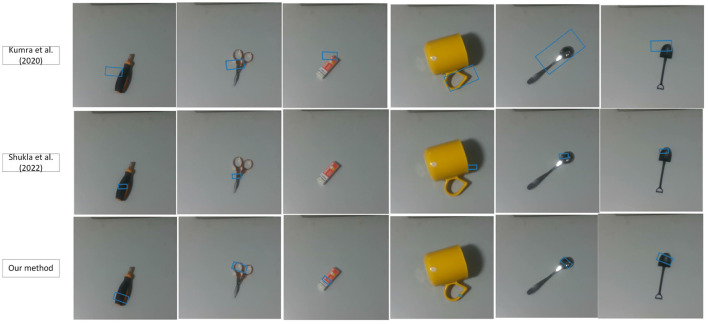
Untrained single tool images.

**Figure 7:**
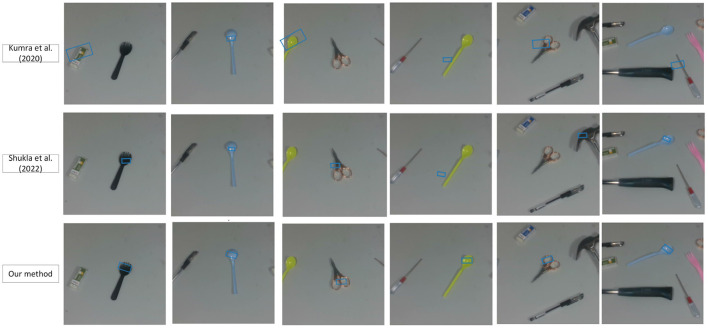
Untrained multi-tools images.

Considering that the traditional method does not involve the content of the state task area, we regard the entire object as an area with a grasp attribute, that is, the mask is the entire tool. We compared the accuracy of our network with the results of previous experiments on the Jacquard dataset (as shown in Table 3). It can be seen that the more accurate what needs to be captured, the more obvious the superiority of our method is. To further test the effectiveness of our grasping network, we tested it on a dataset of tools constructed by ourselves. Tool images are captured by a realsense camera. It is worth mentioning that our training set does not contain images from our homemade dataset. We have compared with Kumra et al. (2020) and Shukla et al. (2022), as shown in Figures 6, 7. It can be seen from the Figure 6 that in the untrained real images with uneven lighting, our method can more accurately find the grasp configuration of objects, and adopt a suitable size of the grasp box. For example, when grasping a cup, a small frame is generated at the handle of the cup to avoid the collision between the gripper and the rest of the cup. Figure 7 shows the strong anti-interference ability of our method and proves the necessity of generating object mask.

**Table 3:** We compared our grasp network with other work.

**References**	**Accuracy (25%)**	**Accuracy (50%)**
Depierre et al. (2018)	0.74	–
Zhou et al. (2018)	0.92	–
Kumra et al. (2020)	0.94	0.72
Depierre et al. (2021)	0.86	–
Shukla et al. (2022)	0.90	0.69
Ours	0.95	0.77

## 5. Conclusion

We presented a modular solution for tool usage issues in the context of human-robot interaction. A multi-layer instance segmentation network helps robots understand the regional attributes and semantics of objects under different states. Based on the state assigned to the robot based on the task, it is able to grasp or handover novel objects using our convolutional neural network MGR-Net that uses *n*-channel input data to generate images that can be used to infer grasp rectangles for each pixel in an image.

We validate our proposed system on our robotics platform. The results demonstrate that our system can perform accurate grasps for previously unseen objects with different state, even our method is able to adapt to changes in lighting conditions to a certain extent.

We hope to extend our solution to more complex object environments, such as where tools overlap and occlude each other. Besides, combining multiple visual angles to improve the success rate of grasping should also be considered in our later work.

## Data availability statement

The datasets presented in this study can be found in online repositories. The names of the repository/repositories and accession number(s) can be found in the article/supplementary material.

## Author contributions

TX proposed the method and designed experiments to verify the method, and then wrote this article. DZ and JY assisted in the experiment. YL reviewed and improved the manuscript. All authors contributed to the article and approved the submitted version.
